# From ‘the global’ to ‘the local’: the future of ‘cooperative orders’ in Central Eurasia in times of complexity

**DOI:** 10.1057/s41311-020-00262-4

**Published:** 2020-07-24

**Authors:** Elena A. Korosteleva, Irina Petrova

**Affiliations:** School of Politics and International Relations, University of Kent, Canterbury, CT2 7NX UK

**Keywords:** Complexity, Cooperative orders, EU, China, Russia, Central Eurasia

## Abstract

Living in times of increasing complexity is hard; it becomes even harder with the realisation of diminishing control. How do we adapt our governance to this complexity to ensure peaceful cohabitation of the established and emergent order regimes? This paper contends that it is important to embrace complexity in full, conceptually and practically, by shifting from ‘the global’ to ‘the local’, to understand the pressure of transformational change and to prepare the ground for the emergence of more resilient and cooperative orders. We apply this complexity-thinking, using a 3P analysis, to Central Eurasia, presently a battleground of three competing order-making regimes—the EU, China and Russia. We argue that for more resilient and cooperative orders to emerge, it is essential to understand and enable ‘the local’ and embrace the region in is diversity, to facilitate a more joined-up and bottom-up governance in managing the complexity of a changing world.

To live in times of change is a curse according to a Chinese proverb. To live *with* change *knowingly*, observing its accelerating pace (Beck 1992) and the emergence of what is now known as a VUCA-world—of increasing volatility, uncertainty, complexity and ambiguity (Burrows and Gnad 2017)—may even lead to an ontological crisis, affecting an individual’s sense of order and continuity with the future, their relationships and experiences (Flockhart 2020). However, depending on how one responds to change, living under these conditions could either become a curse or a blessing, and this is what seems to form the operating premise of today’s changing international environment caught between complexity, rampaging crises, diminishing control and rigid instruments for tackling uncertainty. How should one respond to change to make it work for both individuals and global orders, to make the uncertain future more manageable?

‘Taking back control’ in times of complexity (and crisis) seems to be a popular action these days taken by a range of governments around the world, in an effort to secure their authority and disconnect from global challenges to nurture national survival in isolation. Its long-term benefits, however, are uncertain and highly controversial, as the COVID-19 pandemic has vividly demonstrated.^1^ While shutting borders and imposing central control may have helped to contain the virus short term, coping with it and exiting the pandemic lockdown would require a far more complex and collective action than individual governments could afford on their own (Tocci 2020), given the ‘inherent dynamism, and connectedness of the modern world’ (Korosteleva and Flockhart 2020, p. 160). If anything, these insulating strategies are likely to bring about even more uncertainty and fragmentation, making these nationalist scenarios unsustainable.

The other option is to ‘go global’ and expand existing hegemonic orders, which are often seemingly legitimised on the grounds of their historical longevity, and claims to normative ‘universality’ and inter-cultural affinity (Smith 1996). The premise of this logic is to export ‘rule-/value-based order’, to make the external environment behaving ‘like us’ and aligning ‘with us’, this way also hoping to extend one’s authority and also prevent importing threats (Diez 2005). While there is nothing wrong with engendering ‘like-minded orders’, their expansion to date has often resided on a one-way conditional co-optation generating dependency instead of healthy competition (e.g. China–US current relations) or, in more extreme cases, military intervention (e.g. Crimea; Iraq; Libya, or Iran more recently). With the advent of a multi-order/multiplex world (Flockhart 2016; Acharya 2018) and further redistribution of wealth and resources, this option, too, proves unsustainable. This is manifest, for example, in a crisis of liberal international order (Mearsheimer 2019; Ikenberry 2018; Duncombe and Dunne 2018), the upsurge of populism and ‘petty trade wars’ between the established and rising powers (Cox 2017).

A third option, as advocated by this article, is actually to embrace complexity in full, both conceptually and practically. This means moving from ‘the global’ to ‘the local’ but *not* to disconnect (as the first option implies), rather—to understand change and its effect on ‘the person’ locally, and connect it back to ‘the global’ by enabling collective *resilience* and facilitating *cooperative orders*, as was posited by the European Union’s (EU) Global Security Strategy (EUGS) in 2016. For this to happen, the foundations of how the world is governed today must be rethought, both in theory and practice. As Kavalski argues, one ought to make complexity-thinking an integral part of International Relations (IR)—Complex IR (CIR), which is the conceptual premise of this article—to advocate for a new ‘vision of politics that emphasises responsibility’ and ‘immanent self-ordering’ (2007: 450). This, in turn, implies a new way of bottom-up governing, enabling ‘the person’ (as a collective) to handle change and actualise their potential the way *they* specify, which would be inclusive of a people’s sense of ‘good life’, their identity reflected in power projections, and the principles and practices to guide daily behaviour—this way, forming foundations for the emergence and co-existence of new orders.

Applying this new thinking to understand how the newly emergent orders may co-exist would be particularly relevant for such complex geography as ‘Central Eurasia’, spanning Belarus in the west to Tajikistan in the east and the Caucasus in the south.^2^ This geographical space has been besieged by power struggles for order-making initiatives between the EU, China and Russia, to name but a few, who in their zeal to extend influence often fail to acknowledge not just the sheer complexity of this historically vibrant region, but also its multiple voices and the need for cooperation if more sustainable orders were to occur.

This article will deploy a ‘three-P’ (3P)—power, principles and practice—framework, developed by Flockhart (2020) as better suited to understand governing challenges when engaging with such a complex and diverse region as Central Eurasia. Notably, we will examine if a reflective shift in governance from ‘the global’ to ‘the local’ by the three lead powers is really happening, to establish a more cooperative environment in response to complexity, and develop a shared learning bottom up. The latter is particularly important as it is supposed to reach out to peoples’ ‘hearts and minds’ to help unlock their potential for a more resilient future in the face of complexity and crisis. Applying a 3P analysis should allow us to go beyond unpacking resilience as a new governing thinking (self-governance), developed elsewhere.^3^ Instead, this article aims to look specifically at the nexus between *power* rhetoric, based on *principles* and *actions* by the three entities vis-à-vis the region to understand if more resilient and cooperative order(s) are possible across Central Eurasia. To this end, we will examine the three actors’ power projections, normative promises and practices to demonstrate how currently disjointed and disconnected their visions and actions are in relation to the region, and vis-à-vis each other. This may suggest that even before any resources could be committed and infrastructures built, power strategies ought to engage with the ‘local’ and each other, to embrace *complexity* more fully in the development of more sustainable orders.

The article will proceed by first, briefly introducing the 3P framework and then applying it to each chosen actor vis-à-vis the Central Eurasian region. It will conclude with further discussion of the ‘missing elements’ in today’s global strife to develop more adaptive governance strategies for managing complexity, especially with the rise of local visions across Central Eurasia.

## A 3P framework for embracing complexity

Before we proceed to examining the interplay of competing strategies by the EU, China and Russia over the region in order to ascertain complexity challenges for the emergent order(s) there, it is essential that first, we introduce the fifth IR debate, or ‘Complex International Relations’ (Kavalski 2007) as our conceptual premise, and posit the argument about the *relational nature of complexity* (Qin and Nordin 2019) to understand change, and second, we introduce a 3P framework developed by Flockhart (2020), to help comprehend how to deal with the growing complexity in the Eurasian region.

By urging to add complexity to IR as a discipline to change our way of thinking, Kavalski (2007) posited the advent of a so-called fifth debate in the study of international life.^4^ Compared to the established IR theories, ‘Complex IR’ (CIR), underpinning the new Grand debate, rests on different ontological and epistemological premises. Ontologically, it rejects ‘the assumption of traditional IR that ‘in any particular context, at any particular time, there is a single reality out there waiting to be discovered’ (Kavalski 2007, p. 446). On the contrary, developments are seen as nonlinear, emergent, constantly adapting and self-organising. In this sense, the ontology of CIR is close to the *relational turn*, as ‘relationalism represents an attempt to recast social ontology in a way that rejects essentialist notions of social units’ (Emirbayer 1997) and sees social transactions and processes as the fundamental constituents of social reality’ (Bousquet and Curtis 2011a, p. 49). Epistemologically, CIR is built on the premises of bounded rationality, implying that even the most advanced research methods will provide only limited understanding of social phenomena. These foundational assumptions have important implications both for academic and practitioner communities, when dealing with a VUCA-world.

As Grove and Chandler note in their work (2016), complexity is omnipresent and increasing, manifested in the arrival of the Anthropocene, that forces governments and scholars alike to develop new thinking to understand its unpredictable and emergent nature—all with a purpose to find solutions to managing the uncontrollable effect of complexity. The EUGS rightfully reflected on these developments by stating that we were faced with a rapidly changing global environment, which became ‘more connected’, ‘more contested’ and ‘more complex’ associating with ‘global power shifts and power diffusion’^5^ and a world of ‘predictable unpredictability’ (2016, p. 46).

However, simply acknowledging complexity, as a growing body of literature (Geyer 2003; Kavalski 2007) and policy-makers (EUGS 2016; World Bank 2016; Tocci 2019) have done, is not enough. It is essential that scholars and practitioners embrace it in full by embedding it in their thinking and action, so that it forms part of govern mentality to be able to better respond to change—for it to become a blessing rather than a curse. Hence, while recognising that complexity is marked by the processes of nonlinearity, relationality, unpredictability, self-organisation and emergence (Bousquet and Curtis 2011b; Luckmann 1990), we also need to learn to manage it bottom up, as it ‘cannot be regulated… but [only] experienced’ (Kavalski 2007, p. 450).

It is essential to begin reshaping the foundations of scholarly thinking through action in order to manage the uncertainty of the future more efficiently. This could be done, first, by shifting the focus on to ‘the local’ to understand the workings of what Flockhart (2020) calls, a locally constituted (ideal-type) ‘ordering domain’—of communities, societies and states—to see how they would cope and adapt to change, and second, by connecting it back with ‘the global’, to determine what kind of support (and not by way of intervention, co-optation or coercion) is necessary to ensure more resilient and cooperative response when dealing with a less predictable future. While growing resilience as self-governance constitutes an important part of managing complexity (Korosteleva and Flockhart 2020), this article, however, more specifically looks at the ongoing projections of power by three lead actors (the EU, China and Russia) based on their principles and practices, in the process of (re)forming regional and international order(s). The underlying question is whether in their projections of governance, they can actually instigate sustainable order(s) to ensure a more resilient and cooperative response to managing complexity.

As Flockhart contends (2020), the purpose of introducing an ideal-type ordering domain with its origin in ‘the local’ is precisely to provide us with a more unifying construct for analysing how communities, societies or states respond to change, and how orders emerge and interact, in their evolution and response to complexity. The 3P framework in particular, inclusive of power, principles and practices of operation for any-level ordering domain, does not only lay the foundations for a comparative overview of more/less effective governance strategies locally. It is important for starting a discussion about engendering new cooperative architecture to support emergent and existing orders, in response to complexity and change.

The* power element* of an ordering domain, as Flockhart claims (2020, p. 221) on the one hand, is characterised by the type of authority and hierarchy present as well as the notion of identity and a sense of ‘the good life’ that come to underpin it and bring communities and societies together.^6^ On the other hand, it also relates to how this domain tends to extend its *authority externally*, driven by the notion of self-worth and its perception of the outside/‘the Other’ as a way to validate its own ‘way of living’, to bring stability and minimise threat (Nicolaidis 2016). Here, the narratives of identity, ‘good life’ and ‘shared norms/values’ come to the centre stage of the external power projections, especially in terms of how an actor uses them—via co-option, coercion or cooperation—to exert governance over the outside and to ensure the stability of its own domain in managing complexity. As Korosteleva and Flockhart (2020) argue, for any ordering domain to survive, it would have to be *reflective of* and responsive to the changing external environment, to find ways to maintain the system’s equilibrium and to be able to exert influence. We will explore this element in each actor’s case later in the article, to demonstrate how *reflective and yet, un*-*relational* the projected visions and ways of delivering them are in each given case, and how distant they are from the needs and expectations of the local communities.

The* principles element,* Flockhart contends (2020, p. 223), refers ‘primarily to the ideational structure of the domain expressed through its shared values and a sense of “good life”’. It represents an assemblage of norms, values, rules and visions that a community of a given domain largely shares including its practice. The extent to which people adhere to these ‘principles’ and values of their ordering domain also reflects the latter’s cohesion and legitimacy, as well as their ability to adapt and extend them externally, through shared learning. Although all ordering domains from time to time go through the moments of contestation and soul-searching against the established conceptions of ‘the good life’, as Flockhart claims (Ibid), some prolonged manifestations of dissent should be taken seriously. A shared conception of ‘the good life’ is not just essential for domain’s internal stability—it is also instrumental for its capacity to transform and have a relational value for ordering processes.

Finally, the* practice element* is more about institutions and agential behaviour of upholding and engaging with norms and rules of the domain. Externally, they serve the purpose of legitimation of one’s authority, to establish access to and dialogue with the outside. As Flockhart argues (2020, p. 224), ‘[institutions] serve a dual function of reinforcing the identity and power patterns of the ordering domain through a performative expression of norms, values and the vision of the “good life”, and providing services necessary for fulfilling objectives of the ordering domain’. While institutions must offer support and cognitive consistency among most members of the ordering domain, they too should have the capacity to cooperate, especially in a complex environment. The only way ‘normative practice’ could be seen as legitimated externally is if it were to appeal to the ‘hearts and minds’ of other communities by generating their ownership of the process. Hence, overcoming internal/external duality with the purpose of developing a ‘shared normal’ becomes a prerequisite for the rise and stabilisation of separate ordering domains, and their subsequent dialogue—a precursor of a more adaptable and cooperative outside.

It is worth noting that the 3P elements in this analytical framework are inherently interlinked, and should not be studied in isolation. At the same time, each focuses on a specific question, to make the inquiry distinct analytically: the first is about how *reflective* a given actor is of its external environment to be able to adapt to it; the second is about how *relational* an actor is in being able to share norms/principles with the outside and learn from it; and the third is about how *capable* an actor is, using its reflective and relational experiences, *to legitimise its presence,* transform and develop cooperation with others. We shall now see how this analysis could aid our understanding of the emergent (order) complexity of Central Eurasia, and the degree of relationality between the ordering initiatives of the EU, China and Russia vis-à-vis the region, and each other.^7^

## The EU resilience as a new regime of adaptive governance?

The EU has come a long way in spearheading its order-building initiatives especially towards the eastern neighbourhood. Its *modus operandi* there has progressed from standard Partnership and Cooperation Agreements (PCAs) of the mid-1990s to more advanced and differentiated forms of cooperation—the Association Agreements (AAs)—since 2014 under the European Neighbourhood Policy (ENP). The latter can now provide some hitherto unthinkable sectoral opt-outs as is in the case of Armenia’s Comprehensive and Enhanced Partnership Agreement (CEPA) and ongoing tailored negotiations with Azerbaijan and Belarus.^8^ For the past few years, the EU separately worked hard to re-launch a new Strategy towards Central Asia, which came to replace its outdated and ill-equipped vision of 2007 (Korosteleva and Bossuyt 2019). Clearly not all governing efforts have been successful, which forced the EU to keep modifying its external strategy (Nilsson and Silander 2016; Oskanian and Averre 2018; Bossuyt 2018). This, on the one hand, testifies to the EU’s *reflective* thinking vis-à-vis the region. On the other hand, however, judging by a varied (and largely tepid) regional response (Korosteleva et al. 2020), many of the complexity challenges still remain. It may be that while being reflective about its governing instruments, the EU is still too centred on itself, positioning as a ‘pole of attraction’ for the European way of living, and a ‘hub’ for legal international rules and norms (Delcour and Wolczuk 2017). We will examine these challenges using the 3P framework below.

## The power element: a shift to more adaptive governance?

Our tentative analysis of EU relations with the region suggests that there have been at least three reflective shifts in EU thinking to make its governance more effective and adaptable. The first shift associated with the launch of the ENP as a new framework for extending EU influence outside. Given the enlargement success in transposing its power, principles and practice, the EU tried to apply this model (albeit without a membership prospective) to its immediate neighbourhood. This resulted in the adoption of so-called *‘disciplinary governance*’ (Korosteleva 2018), premised on a largely hierarchical bilateral mode of coordination and prescriptive (by conditionality) agenda setting. This was clearly an EU-centric, one-sided approach to exporting the EU order beyond its borders, which anticipatedly yielded limited response from the region (Casier 2011; Börzel and van Hüllen 2011).

To improve its credibility, the EU went through a series of further iterations. Initially, governance actions included further regionalisation (2007/8), and differentiation to seek consultation and partnership with different-level stakeholders via a ‘more for more approach’ (2011). This new EU approach could be described as *‘deliberative governance’*, associating with the recognition of partners’ interests at the negotiation table, developing a dual track engagement with multiple stakeholders, and less interference in the implementation process. The EU also included regular consultations with policy stakeholders, and yet, the response was almost similar to that of the previous years in terms of reciprocation, with the exception of Ukraine, which unexpectedly became a battleground of openly contested ordering initiatives between the EU and Russia. The subsequent annexation of Crimea and the ongoing crisis in Ukraine serve as a reminder of still largely mono-logical actions by the power actors failing to understand the underwriting complexity of the wider region.^9^

The EU has now concluded another round of consultations in an attempt to align its wider neighbourhood (now also to include Central Asia) policy and action plan with that of the EUGS 2016, with resilience taking a centre stage. It is argued elsewhere^10^ that this shift in EU thinking, tentatively named as ‘*adaptive governance*’, is important for testing the EU’s ability to be a reflective (and effective) order-making actor in Central Eurasia. On the one hand, the EU’s intention, at least in rhetoric, is to decentre from its own agenda, to the level of partner states and local communities, to better understand their needs, and enable them to seek their own solutions to development problems. This should, in theory, enable individuals to grow their strength and capacity to better manage change via local transformation, and where necessary, with the external support (and not intervention) of the EU—just as Kavalski (2007) imagined what CIR should do when embracing complexity.

On the other hand, there are already some warning signs to show that ‘business as usual’ continues, especially when it comes to instrumentalising ‘local ownership’ for the purpose of legitimising the EU’s own agenda in managing external insecurities (Petrova and Delcour 2019). Perhaps it may be too early to evaluate this new turn in EU governance. However, the way the EU comes to practise resilience—by way of bringing its own solutions to local problems, and treating them as risk and security vulnerabilities for its own environment (Korosteleva 2019)—seems to tell us a familiar story of the hitherto non-relational, EU-centric engagement with the wider region.

The same seems true for the level of EU strategic partnerships with Russia and China, vis-à-vis the region. While respective bilateral platforms have been set up, they fall short of progress in relation to resolving some complex regional issues. Notably, the EU–Russia platform largely remains non-operative due to EU-imposed sanctions that followed the annexation of Crimea (Bossuyt and Bolgova 2020). The EU is slow to respond to other emerging initiatives that may give it better traction in the region—e.g. the Eurasian Economic Union (EAEU), which, despite being almost a decade old, is still not recognised by the EU contrary to EU business lobbying (Schneider 2019). The EU–China dialogue vis-a-vis the region is also limited, especially (and paradoxically) in linking the EU TEN-T agenda^11^ to the Chinese Belt and Road Initiative (BRI), which purportedly aims to connect Europe and Asia on many different levels. In short, the EU is doubtless *a reflective actor* in trying to improve its governing outreach across Central Eurasia, and yet, as the next sections reveal, it has still to learn to become a relational, legitimate and cooperative player to make its governance effective for managing complexity.

## The principles element: more rhetoric than action?

While being reflective about its governing instruments, the EU clearly falls short of opening up its rule-based order to learning from external normative experiences. To be fair, it has progressed in its discourse of accepting that a European way of living, if not ‘universal’, is still very competitive to become a pole of attraction and stability. Since the Maastricht Treaty and its enlargement, the EU has developed a coherent identity narrative for sharing it externally. There is a strong belief that the EU core values of liberty, peace, democracy, human rights, rule of law, anti-discrimination, etc. (Manners 2002) are universal in nature and should act as an anchor for a century-long experience of norm promotion by European powers.

Eurocentrism today has strong historic roots and rests on multiple sources of legitimation. For instance, the EU has been successful in advancing a narrative that removed its colonial past (Lagrou 2009; Bhambra 2009; Hansen and Jonsson 2012). At the same time, it managed to construct a sense of continuity between Europe from Antiquity and the EU as the end-point (Swedberg 1994; Dussel 2000). Other sources of legitimation include the myth of origin as a peace project and a progressive modernising force (Ifversen 2010), as well as a search for unity which has been inherent to the ‘habits of thought and practice’ (Pasture 2015, p. 202) of the European states for centuries. As Pasture argued, diversity was traditionally perceived as dangerous because Christendom ‘contained an ideology of homogeneity, considering difference as detrimental to the godly order, which was much reinforced through the association between worldly and religious power’ (Pasture 2015, p. 199). These deep-seated foundations of Eurocentrism coupled with the triumph of liberalism in the Cold War and the success of the EU enlargement policy, resulted in the EU’s self-perception as a universal normative power (Manners 2002), putting promotion of its norms at the heart of its external action, particularly so in Central Eurasia.

However, lately the EU seems to have begun opening up to other norms, at least rhetorically. As the EUGS stated, ‘we will not strive to export our model, but rather seek reciprocal inspiration from different regional experiences’ (EUGS 2016, p. 32). However, the same document also indicated that ‘in the pursuit of our goals … we will work with *like*-*minded* countries and regional groupings’ (Ibid, p. 8; emphasis added), thus once again suggesting anchoring new partnerships in the EU-based vision of order.

## The practice element: hard to implement?

Putting reflectivity to practice proves much harder. Having pioneered the people-to-people dimension in all its ENP sectoral policies as early as the 2000s, the EU still struggles to make its ‘hearts and minds’ appeal more tangible (Schumacher et al. 2018; Petrova and Ayvazyan 2018; Ahrens and Hoen 2019). The relatively new ENP Instrument (ENI), while allowing a range of measures for bottom-up local capacity building (Korosteleva 2019), only offers five-per cent budgetary support for local initiatives, while the remainder goes to support pre-set planning and target management (Ibid, p. 21). Much of the new partnership negotiations agenda in the region is still EU centred—that is, despite the EUGS commitment to act by ‘principled pragmatism’ and in the interests of all parties. This discrepancy is also reflected in EU dwindling relevance across Central Eurasia, although there are signs of the emergent new interest (Bossuyt and Bolgova 2020).^12^

The EU actions are yet to support its vision of cooperative orders and more responsive global architecture. One example, briefly mentioned earlier, is the EU reluctance to engage with the EAEU. As Marciacq and Flessenkemper (2018, p. 11) note, ‘while usually support[ing] interregional dialogue worldwide, the EU has not explicitly recognised the E[A]EU and only conducts limited dialogue with this counterpart at the technical level’. There may be a range of rational explanations to justify such a choice. However, this practice explicitly goes against the EUGS professed commitment to facilitating cooperative orders and engaging with regional complexity. Furthermore, the unintended consequence of non-engagement also deprives the EU of opportunities to leverage Russia, and more so, the EAEU’s smaller states, who, in their institutional expansion, look up to the EU as a model.

Another example (out of many) is the EU continuing struggle to maintain the trialogue between the EU, Russia and Ukraine, to seek crisis resolution in the region. This is manifest, for example, by the recent negotiations regarding the post-2020 arrangements for the transit of Russian gas to Europe via Ukraine, impeded by EU insistence on adopting its own regulatory framework.^13^ The above instances indicate disjuncture between EU rhetoric and practice, with the latter still heavily grounded in the EU’s prevalent sense of exceptionalism. This may be due to its excessively slow operating machinery when it comes to tying its practice to vision. However, if this stems from not understanding complexity and the region, then the EU, as President Macron recently noted, is likely to find itself ‘on the edge of a precipice’, with no turning back.^14^

## China’s connectivity agenda and its global appeal?

China, too, is grappling with the unravelling VUCA-world, but seemingly in a more adept way, even in the times of the global pandemic. In 2013, the Chinese Communist Party articulated its new external strategy of embracing complexity as ‘One Belt One Road’ or the BRI for external use, which embraces both the Silk Road Economic Belt and the 21st-century Maritime Silk Road (Yang 2019).

By 2015, the BRI policy framework consolidated into the ‘Vision and Actions’, with the State Council’s authorisation, and the Leading Group was established to ‘build a global community of shared future’ (2019, p. 9). We shall now examine the core tenets of this initiative, by looking at China’s relations with Central Eurasia, using a 3P framework, to see, as in the EU case, how reflective and relational its engagement with the region is, and projected to be.

## The power element: cooperative but not relational?

The BRI in a sense is China’s response to the increasing uncertainty, fragmentation and diminishing control over both the internal and external environment. As Jinny Yan, China’s Chief Economist for the BRI, insisted, it is designed to be a non-hierarchical and bottom-up engagement with all-level stakeholders, to develop their resilience and connectedness in the face of future challenges,^15^ which is very much in line with the EU’s own way of thinking post-EUGS 2016. However, China seems to go about it in a much more concrete and determined way.

According to the BRI founding document (Vision and Actions 2015, p. 3), it is supposed to:

promote the connectivity of Asian, European and African continents, and their adjacent seas, establish and strengthen partnerships among the countries along the BRI, set all-dimensional, multi-tiered and composite connectivity networks, and realise diversified, independent and sustainable development in these countries.

It rests on five principles of peaceful co-existence, in line with the principles of the UN Charter (Ibid), and sets to pursue five specific cooperation priorities to ‘boost [multilateral] cooperation platforms’ for ‘increasing mutual understanding, reaching consensus and deepening cooperation’ (Ibid, p. 12).

Cooperation priorities, as identified in 2015, focus on (1) better policy coordination; (2) development of infrastructural connectivity; (3) promotion of unimpeded trade; (4) enhancement of financial integration for better access to investment and resources; and (5) people-to-people bonds. In 2019 priorities were extended to include (6) industrial cooperation for China’s investment in BRI countries. The key ‘cooperation mechanisms’ initially prioritised bilateral links (through joint committees) with the BRI governments (very similar to the EU governance of pre-2011) and mobilise existing multilateral mechanisms, such as Shanghai Cooperation Organisation, ASEAN Plus China (10+ 1), Asia–Pacific Economic Cooperation, and others (2015, p. 9). These mechanisms were supposed to ‘support local authorities & general public of BRI countries to explore their historical and cultural heritage’, thus specifically underscoring the salience of bottom-up governance and cooperative initiatives (Ibid p. 10).

Having celebrated 5 years since inception, the BRI has enjoyed enormous progress in terms of its expansion and development of international cooperation, seeing some tangible results along the way. By the end of March 2019, Chinese government signed 173 cooperation agreements, with 125 countries and 29 international organisations, having expanded through Asia to Europe and Africa, and also procured new partnerships in Latin America and the South Pacific (2019, p. 6). Every year the progress is monitored through consultation and evaluation discussions for further improvement. This way, once again, it resembles the EU effort to stay reflective over its action in realising its objectives. The scope of consultation, however, is different and fully in line with the Chinese ambition to achieve ‘global consensus’ (Ibid, p. 7). For example, with the intention to set up international fora, the first extensive consultation was held in May 2017 in Beijing involving over 16000 representatives from over 140 countries, and 80+ IOs, and producing 279 deliverables with 76 major items in five key areas, which as the official review states have now been implemented (Ibid).

However, the key to this progress is not just about staying reflective and cooperative. Success undeniably rests on advancing the ‘people-to-people’ dimension, which admittedly has been the weakest point of the BRI to date and which has become the centrepiece of BRI 2.0, just like the concept of resilience in the EUGS (2016). In this regard, the key priority for BRI 2.0 lies with ‘establishing a track two mechanism for dialogue’ (2019, p. 8)—to appeal to the ‘hearts and minds’ of the people involving civil society and various political organisations—parties, parliaments, think tanks, local authorities, business communities, the media and institutions for higher learning. With this new thinking, Chinese government is clearly on a steep learning curve to understand the workings of civil society which it has never done before, but as one interviewee commented, ‘we are very quick and committed learners’.^16^

In addition to having bilateral engagement with BRI countries from Central Eurasia, China seems equally open to cooperating both with the EU and Russia as power contestants, vis-à-vis the region, often ceding the agenda-setting initiatives to partners. While the EU response has been cautious, Russia instead chooses to pursue the politics of ‘sopryazhenia’—not aligning but moving in parallel^17^ (Ryzhkov 2019)—in an attempt to establish functioning relations with China (Bossuyt and Bolgova 2020).

## The principles element: towards a shared or Chinese-led learning?

So, what has worked thus far in terms of developing traction and ensuring shared learning also to include a Chinese ‘way of living’, which as the official review document suggests, is premised on ‘the principles of extensive consultation, joint contribution, and shared benefits’ (2019, p. 7)?

‘The people-to-people’ dimension, while being under-invested, has nevertheless seen some interesting results. Initially, for example, the intention to win ‘hearts and minds’ of the populations, in Central Eurasia, was intended via school cooperation and student exchange (a strong EU measure). China put aside funds to invest up to 10,000 scholarships per year for BRI countries and provide financial support for Chinese cultural events. This commitment, as the 2019 review reports, has exceeded manifolds. Investment was granted to support diverse forms of cultural exchange, including Chinese and local festivals, bazaars and art fairs. In 2017 alone, about 40,000 scholarships were issued to students from the BRI countries, to study in China, which is about 66% of all students receiving such scholarships (2019, p. 6). Memoranda of understanding were signed with 24 BRI countries on degree recognition; 152 Confucius institutes and about 149 Chinese language classrooms were set up in 54 BRI countries. Chinese Academy of Sciences for the first time opened its doors to 5000 students at the MA and Doctoral levels from BRI countries. China also issued $2 billion in emergency food assistance; launched 100 Happy Homes projects for the poor and homeless, 100 anti-poverty and 100 health recovery projects across the BRI countries and Central Asia.^18^

This is not a small amount by any measure, even though it may seem negligible compared to the levels of investment to the other five BRI priorities (Yang 2019). The people-to-people dimension is tangible for the communities and would normally work towards disseminating Chinese values and legitimising BRI expansion. This dimension is now seen by the Chinese government as a top priority for BRI 2.0, first of all, to mitigate the top-down nature of the BRI and develop a sense of local ownership; second, to fight corruption that undermines the BRI’s global appeal; and finally, to connect communities for more cooperation and greater sustainability, as the review document claims (2019).

In a nutshell, what China seems to have succeeded in accomplishing in Central Eurasia in less than 5 years has taken the ENP its lifetime. And yet, how different are their ordering practice and governing outcomes comparatively? Let us examine the practice element of the BRI.

## The practice element: much ado about nothing?

The EU approach has been reflective about its external governing strategies learning to focus, through resilience, on empowering local communities, but struggling to decentre from EU norms, and EU way of living. Conversely, the Chinese model seems more adept and practicable in its pace and penetration, although still working top down, and until now, unable to engage with civil society to develop a more sustainable *modus operandi*. The two powers also differ in the grandeur of their visions, and China by far outpaces the EU in its ambition to become ‘global’, conceptually and practically, working through different stakeholders.^19^

And yet, despite these differences, the two powers are very similar in how they protect and project their way of living and norms. China, for example, invests heavily in education, but in its own country—by bringing BRI students in and capitalising on ‘brain drain’ exiting the Central Eurasian region. The EU has already passed that phase, gradually beginning to invest into local infrastructure to turn ‘brain drain’ into ‘brain circulation’. China brings its own labour to the new markets, for job opportunities and capital production; the EU brings its own goods, by setting up PCA and AAs for this matter. Both powers operate via co-optation: China through ‘easy access money’; the EU—through conditionality. Confucian institutes infiltrate every country along the BRI; Chinese language and cultural awareness are growing, just as the Europe Direct initiative used to operate across Central Europe, and English has become the dominant tongue. In other words, both powers are seeking to promote their way of living, often at the expense of the local, and their (possibly different) sense of ‘good life’, ambitions and needs. This is what has been causing disquiet and resistance and in some cases even outright rejection (Cavanna 2019:30; Murphy 2019, p. 4).^20^ In summary, while governing objectives of both powers may be reflective of change and the need to go ‘local’ to understand and give more voice to the region; practice, at least for now, speaks of the opposite, and public resistance serves as testimony to both powers’ failure to develop traction and legitimation, let alone—encourage ‘self-governance’ of local communities to actualise their own potential in their strife for ‘good life’ the way they specify.

The two powers at least talk to each other, through the established EU–China platform (Bossuyt and Bolgova 2020).^21^ Yet, this platform does not involve the region as independent actors that both powers target for development, which is another testimony to the narrowness of these ambitions, which in the world of complexity are tantamount to a de facto failure, and inability to change.

## Russia’s policy of ‘sopryazhenie’^22^: aligning, order-making or sitting on the fence?

Having always considered the eastern neighbourhood ‘common’ as opposed to ‘shared’ (Shishkina 2013), Russia has been hard at work arranging its own ordering domain across Central Eurasia, focusing most notably on its institutional and ideational structures. In what follows, we analyse these structures, to reflect on Russia’s governance strategies in Central Eurasia and its interrelations with the two other ordering domains.

## The power element: new strategies, old practices?

There is a clear understanding among Russian political elites that international processes today are complex and nonlinear, with a high degree of unpredictability often requiring urgent response and preparedness (MFA 2013; Putin 2019). Over the past decade, Russian governance strategies in its neighbourhood have gone through a series of changes to better manage the increasing complexity of intra-regional relations. One such important signifier was the decision to abandon the idea of a wide-scale integration across Central Eurasia and instead focus more on thematic projects—e.g. an ‘EU-modelled’ trade bloc (Keukeleire and Petrova 2014). Consequently, the Customs Union between Belarus, Kazakhstan and Russia created in 2010, soon grew into the EAEU by 2015. As compared to the previous practice of ‘virtual’ or declarative regionalism (Allison 2010), the EAEU stresses member states’ commitment to actual (economic) integration, to advance the union of parity and peoplehood. In 2011, Putin argued:

I would like to emphasise that it is highly important for us that the general public and business communities in all three countries perceive the integration project not as some kind of wheeze orchestrated by the top bureaucracy but as a living organism, and as a good opportunity to implement initiatives and succeed.

While still developing, the EAEU has already encountered much criticism for its Russia-centric nature, and a rather coercive/hierarchical style of management and decision-making (Dragneva and Wolczuk 2013).^23^ This suggests that while embracing complexity, it is the hegemonic rather than cooperative intentions that continue to guide Russia’s external governance.

Another sign of adapting to complexity was Russia’s engagement with the concept of soft power, which entered the Foreign Policy Concept in 2013. While historically Russia’s cultural influence has been strong across Central Eurasia, since the early 2010s it received a new momentum. The objective was to exert a form of *disciplinary governance* over Central Eurasia by using soft power means, including the inherent cultural affinity with the region and appealing to people directly. To this end, a broad range of resources and mechanisms was developed, e.g. the Direct line with the President, Russkiy Mir Foundation, the Federal Agency for the Commonwealth of Independent States, Compatriots Living Abroad and International Humanitarian Cooperation (Rossotrudnichestvo), the Alexander Gorchakov Public Diplomacy Fund, Friendship Society etc. (Forsberg and Smith 2016, pp. 131–132).

However, while these policy changes may demonstrate a degree of a *reflective approach,* similarly to the EU’s and China’s, ‘old’ practices of one-sided and Russia-domineering relationships still dominate. These are often associated with both hierarchical and horizontal (‘people-to-people’) use of power where rhetoric often contradicts practice. For example, while reiterating voluntary membership—‘we are not going to hurry up or nudge anyone. A state must only join on its sovereign decision … [and] long-term national interests’ (Putin 2011)—in practice, it is frequently combined with coercion and involuntary co-optation, as was in the case of Armenia (Ter-Matevosyan et al. 2017) and Belarus (Dragneva and Wolczuk 2013) which were pushed to join the EAEU. Horizontal types of co-optation include current negotiations with Uzbekistan concerning its EAEU membership^24^; and Belarus vis-à-vis Russia’s renewed zeal in pushing for deeper integration within the Union State. In other words, the Russian strategy of soft power remains firmly embedded in Realist thinking, seeing soft power resources as just another instrument in hegemonic power bargains, rather than an opportunity for bottom-up cooperation (Nye 2013).

Concerning Russia’s relations with the EU and China, Russia presently is on a turf war with the EU^25^ and strategically mindful of China. Interestingly, its position towards the BRI could be described more as a policy of ‘sopryazhenie’ rather than alignment, contrary to its rhetoric of ever-closer partnership with China (Bossuyt and Bolgova 2020). This raises questions about Russia’s professed commitment to regional alignment—be it vis-à-vis its strategic partners or with the wider region—and its understanding of complexity which seems to be erring towards hegemony rather than cooperation.

## The principles element: aspiring normative hegemony in the region?

As was argued elsewhere in this Special Issue,^26^ Russia asserted an alternative order in Central Eurasia, which is characterised by a distinctive set of principles. After a decade of soul-searching in the 1990s, a new paradigm heavily drawing on the Imperial and Soviet legacies had crystallised in the early 2000s. Contrary to the EU, the core of the Russian (order) principles is constituted by survival, rather than self-expression values (Inglehart and Welzel 2005). The conservative turn taken by the Kremlin in the early 2010s found a fertile ground and strongly resonated within the Russian society, *inter alia* due to its rich historical tradition in Russia (Chebankova 2016; Verpoest 2017). The main norms and values underlying this order include the prevalence of security over liberty, stability, sovereignty, strong state and leadership, *étatisme* and, by extension, paternalism.

Essentially, these norms are presented in the Russian official narrative as not simply confined to Russia, but characteristic of a broader Central Eurasia. The official narrative depicts Russian identity as inherent to a ‘wider community’ (Feklyunina 2015, p. 783) or to ‘Greater Russia’ (Nygren 2008; Richters 2013), thus framing it as ‘country-civilisation’ (Naydenova 2016:42) and, consequently, as Russian ‘civilisational space’ (Laruelle 2015; Coker 2019). Hence, the principles promoted in this order are strongly Russian centric and are often viewed by Russian political elites as universal for the region.

While it may seem contradictory, these domestic narratives relate to Russia’s belief in a multi-order world advocating for equality of civilisations and cultures. The leitmotif of many official speeches revolves around respect for different norms and principles based on a dialogue of equals. Curiously, while attempts to promote Western principles in Russia’s ordering domain are perceived as a threat, Russia’s own, often coercive, actions in the region are seen as order maintenance.

In summary, while Russia may be a *relational actor* on a system level (frequently using its soft power-based cultural, historical and geographical affinity to the region), it has also proved reluctant to learn from the norms, traditions and aspirations of its neighbours on the regional level, which implies that facilitation of cooperative orders with its participation is highly unlikely.

## The practice element: old habits die hard?

Similarly, to the EU, Russia’s reflectivity has also proved difficult to translate into practice, with the latter being clearly affected by the established, deeply rooted relations with its neighbourhood. Beside the cases of Armenia and Belarus vis-à-vis the EAEU, there are many other examples to illustrate this argument. Notably, due to Russia’s pressure, much of the EAEU code of practice has been made advantageous for Russia, thus causing concerns and grievances among EAEU member states.^27^ This also relates to the Kremlin’s continued practice of using energy prices and trade suspension as a political tool within the EAEU (Korteweg 2018). In a similar vein, while having a natural advantage in terms of cultural affinity, ubiquitous language and media presence, Russia’s soft power is seemingly failing to fully engage the people-to-people dimension across the neighbouring states (Sergunin and Karabeshkin 2015).

Concerning the facilitation of cooperative orders, Russia in rhetoric has been one of its most vocal proponents. As Putin argued in 2011:

The EAEU will join the dialogue with the EU. Apart from bringing direct economic benefits, EAEU accession will also help countries integrate into Europe sooner and from a stronger position. In addition, a partnership between the EAEU and the EU that is economically consistent and balanced, will prompt changes in the geo-political and geo-economic setup of the continent as a whole with a guaranteed global effect.

In practice, however, over the past decade not only the two regional orders failed to establish a dialogue with one another, and vis-à-vis the region; they drifted further apart to what is now seen as ‘principally incompatible’ orders (Suslov 2016). Russia strongly believes that ‘international order is eroding while international institutions either do not function or serve the objectives of a certain group of states’ (see endnote 23), thus necessitating a shift away from the West, resurgence of Russia’s own order and a quest for new alliances as manifested by a new Asian vector or what Lavrov’s called, a ‘Greater Eurasian order’.^28^ The practice, however, has shown that the politics of ‘sopryazhenie’ even in theory would struggle to create a ‘shared normal’, let alone a ‘shared order’, be it with Asia or with the EU, from Lisbon to Vladivostok.

## Are cooperative orders possible in the emergent complexity of Central Eurasia?

This article started from the observation that *complexity* associated with uncertainty, unpredictability and crisis (Kavalski 2007; Grove and Chandler 2016) is becoming a new normal in international relations, which requires new thinking and governing strategies. Noting different responses to change, such as rising nationalism often driven by a slogan ‘to take back control’, and the expansion of existing hegemonic orders to make the outside ‘like us’, this article has argued that a more efficient way is possible. Specifically, with the rise of the VUCA-world ridden by crises and uncertainty, it is essential to embrace complexity in full by, firstly, shifting the focus from ‘the global’ to ‘the local’ to understand the challenges of adaptation in the context of emerging/existing orders, and secondly, by facilitating more resilient and collective responses to change. As posited by the complexity theory, resilience is a property of a system (but also an analytic of governance) that can only be developed bottom up, and challenges, therefore, must be addressed locally for more response governance structures to emerge. This complexity-thinking was applied using a 3P analysis (power, principles and practice), to Central Eurasia (a geographical locality rather than a political construct) as an embodiment of the intricate interplay of power relations between the EU, China and Russia vis-à-vis the diversity of the region.

As was shown above, all three powers targeting Central Eurasia have been mindful of complexity and adapting their governing strategies towards this multivocal region accordingly. Notably, the EU, Russia and China, to various extent, have been *reflective* of their policies premised on regional response, and tried to adjust to the emergent environment accordingly. They became more adept at differentiating their governance to regional needs, and with time, more engaged with developing horizontal people-to-people relations, each using different approaches and instruments. At the conceptual and rhetorical levels, the EU’s approach has proved most comprehensive, taking its governance, post-EUGS to a seemingly new ‘decentred’ level of engagement with ‘the local’ via ‘resilience’. China initially prioritised bilateral relations with governments and is only now coming to realise that it must foster a bottom-up engagement, for BRI 2.0 to succeed. Russia, in turn, has been using both hard and soft means of power, especially its cultural affinity through language and media presence, to manage the growing complexity of the region more efficiently. And yet, each power, while *reflective*, still centred their governing strategies on their own vision of development priorities for this multifaceted region, and their own understanding of peoples’ needs, thus proving *un*-*relational* to the region’s complexity, and vis-à-vis each other’s initiatives.

All three powers have also introduced and projected elaborate ideational structures, connecting their principles, norms and values with the narratives of ‘the good life’, pertinent to each particular order. As demonstrated by the analysis, despite the rhetoric of a multi-order world and respect for different civilisations, these ideational structures remained strictly self-centred, presenting their own principles as universal for achieving better living across the region. Learning from the norms of others and developing a shared response to complexity proved particularly difficult for the EU and Russia.

Putting reflective power projections and principles to practice, to make respective governing strategies more adaptable and responsive to change, has appeared to be the most problematic. While each power emphasised the rhetoric of cooperation and local ownership, none succeeded in properly connecting with local communities, in a *cooperative way,* to prioritise capacity-building bottom up and inside out. One of the possible explanations is, as Bossuyt and Bolgova (2020, p. 242) argue, ‘the underlying geo-political rivalry between the three actors, as well as their divergent beliefs and approaches to development’. However, while the approaches may indeed be different, what should seemingly unite them all is the pledged effort for connectivity in the context of strategic development goals. The dialogue is slowly emerging at the bilateral level, but multilateral cooperation, especially with a stronger regional voice(s), is still a distant future. It could alter, if in response to uncertainty and change, the leading powers, chose to embrace complexity in full and engage with multiple actors bottom up and directly, rather than just through the financial intermediaries (e.g. EBRD or AIIB). At the very least, this would offer a better insight to how to make governance more effective, and development—more resilient. To live in times of change (and crisis) is a curse; building resilient lives collectively could turn it into a blessing. This would only be possible, if complexity-thinking is fully embraced, turning the future into an opportunity.

## Footnotes

1 A notable example is the UK’s rejection, due to Brexit, of three rounds of EU collective PPE procurement, hoping to outsource the supplies independently. It, however, failed to secure a deal with Turkey, which directly jeopardised the lives of many frontline medical staff and key workers. The Guardian, 13 April 2020.

2 The definition of Central Eurasia in this paper refers more to a *geographical locality* joined by its past experience, rather than a socially constructed region. The locality in this paper excludes China, Russia and the EU, for the purpose of examining the latter’s engagement and governing strategies in shaping the future order(s) across the region.

3 For more information see Special Issue by Korosteleva, E. and Flockhart, T. 2020 (eds.) Resilience in EU and International Institutions. *Contemporary Security Policy,* 41(2). The volume problematises ‘resilience’ as a new analytic of governance to enable the rise of ‘the local’ and ‘the person’ in tackling complexity and the increasing uncontrollability of the outside.

4 For more information see Table 2 on p. 445, of Kavalski’s (2007) paper, outlining five debates in the study of international life: from idealism vs realism of 1920s, through behaviourism, post-positivism, constructivism, to complexity of 2000s.

5 This document preceded the EUGS 2016 and is important for providing background for the new EU strategy to grow resilience and cooperative orders to ensure change and adaptability of global structures http://eeas.europa.eu/archives/docs/docs/strategic_review/eu-strategic-review_executive_summary_en.pdf.

6 Here we adopt this definition of power, as part of an ideal-type ordering domain, deliberately, to underscore the importance of the shift to identity and the notion of ‘good life’ as the core characteristics of power projections, which is different to the mainstream views of power (e.g. 1st debate of IR—with the focus on the material dimension; or of post-structuralism—with the focus on the bio-/population dimension).

7 This article does not intend to offer an in-depth empirical discussion of the three powers’ practices in the region. Rather, its purpose is to contour the discursive positionings of the EU, China and Russia, vis-a-vis the region, to demonstrate the current impossibility of developing more resilient, and cooperative order(s) across this diverse and important geographical space.

8 European Commission. 2020. Eastern Partnership. https://ec.europa.eu/neighbourhood-enlargement/neighbourhood/eastern-partnership_en. Accessed 5 May 2020.

9 See, for example, Sakwa’s, Nitoiu and Passatoiu’s, and Haukkala’s contributions to this volume.

10 See Korosteleva, E. and Flockhart, T. (eds.) 2020 *Contemporary Security Policy*, 41(2), and especially articles by Petrova and Delcour, Anholt and Sinatti, and Joseph and Juncos.

11 See European Commission. 2020. Trans-European Transport Network. https://ec.europa.eu/transport/themes/infrastructure/ten-t_en and the next section on China.

12 For more detailed discussion, see Minsk Barometer, with a comparative overview of EU–Belarus relations and the Eastern neighbourhood response 2017–2019: http://minskdialogue.by/en/research/reports/minsk-barometer-6; Valiyev 2019; on Central Asia, see: Kavalski and Cho 2018; Laumulin 2019.

13 At the time of writing, the negotiations reached full gridlock, which was resolved on 31 December 2019, a day before the expiry of the 10-year contract. For more information on different aspects of relations see https://www.oxfordenergy.org/publications/russia-ukraine-gas-transit-talks-risks-for-all-sides/?v=79cba1185463; and https://www.ft.com/content/5c6733b2-2bc6-11ea-a126-99756bd8f45e.

14 The Economist. 2020. Assessing Emmanuel Macron’s apocalyptic vision. https://www.economist.com/leaders/2019/11/07/assessing-emmanuel-macrons-apocalyptic-vision. Accessed 5 May 2020; The Economist. 2020. Emmanuel Macron warns Europe: NATO is becoming brain-dead https://www.economist.com/europe/2019/11/07/emmanuel-macron-warns-europe-nato-is-becoming-brain-dead. Accessed 5 May 2020.

15 Presentation of BRI 2.0 vision in Vienna and interview with the author.

16 Author’s conversations with Jinny Yan, managing Director & China Chief Economist; Cheng Xie, 1st Secretary, Permanent Mission of China to UN Industrial Development ORganisation; Dr Li Yongquan Director of Centre for BRI (CASS) Beijin; Hao Jiang, Roland Berger in Vienna, 10 October 2019.

17 See Vladimir Ryzhkov’s comments in relation to the BRI: https://m.forbes.kz/process/expertise/odin_poyas_odin_put_stranyi_tsentralnoy_azii_zagonyayut_v_lovushku_dolgov Accessed 5 May 2020.

18 For more information on various aspects of the BRI success in Central Asia, see http://osce-academy.net/en/research/cadgat/.

19 See annual BRI progress reports issued by the Leading Group, Chinese MFA and Ministry of Commerce.

20 For more local accounts about the failure of Chinese effort to connect with people in individual countries, see: https://m.forbes.kz/process/economy/55_kitayskih_zavoda_forbeskz_obyyasnyaet_pochemu_v_kazahstane_etih_predpriyatiy_ne_poyavitsya/?utm_source=forbes&utm_medium=mlt_articles; https://belarusdigest.com/story/china-as-an-epic-failure-of-belarusian-foreign-policy/https://belarusdigest.com/story/myths-of-chinese-engagement-in-belarus-no-money-between-friends/.

21 https://ec.europa.eu/commission/presscorner/detail/en/IP_19_2055; and https://ec.europa.eu/transport/themes/international/eu-china-connectivity-platform_en.

22 ‘Sopryazhenie’ (Russian: сопряжение) can be understood as a degree of coordination of strategies, projects and resources developed under different regional strategies.

23 Russia–Belarus relations are presently undergoing revision, under Russia’s pressure, to revive the Union State, and much closer political, institutional and economic integration between the two entities. See http://minskdialogue.by/en/research/opinions/belarus-russia-where-are-current-tensions-leading.

24 For the discussion including in-country debates, see Hashimova, U. 2019. Not So Fast: Will Uzbekistan Join the Eurasian Economic Union? The Diplomat. https://thediplomat.com/2019/10/not-so-fast-will-uzbekistan-join-the-eurasian-economic-union/. Accessed 5 May 2020.

25 See V. Nikonov’s reflections at the Plenary session of the Minsk Dialogue Forum 2019. https://www.youtube.com/watch?v=MpizBgRzwPg. Accessed 5 May 2020.

26 See H. Haukkala’s and R. Sakwa’s articles in this special issue.

27 In particular, Russia pushed the EAEU to adopt protective Russia’s external tariff, which ensued other states, with very low external tariffs (Armenia, Kazakhstan, Kyrgyzstan) to increase theirs substantially this way contradicting their WTO commitments. The recent spat between Belarus and Russia concerning energy tariffs is also a result of similar hegemonic policies.

28 Lavrov, S. 2019. Neighbours in Europe: Sergey Lavrov about the results of 30 years of cooperation between Russia and the EU. Rossiyskaya Gazeta, No 285(8043). https://rg.ru/2019/12/18/sergej-lavrov-ob-itogah-30-letnih-otnoshenij-mezhdu-rossiej-i-evrosoiuzom.html. Accessed 5 May 2020.
